# The endocannabinoid 2-arachidonoylglycerol promotes endoplasmic reticulum stress in placental cells

**DOI:** 10.1530/REP-19-0539

**Published:** 2020-05-01

**Authors:** Marta Almada, Lia Costa, Bruno Fonseca, Patrícia Alves, Jorge Braga, Daniela Gonçalves, Natércia Teixeira, Georgina Correia-da-Silva

**Affiliations:** 1 UCIBIO, REQUIMTE, Departamento de Ciências Biológicas, Laboratório de Bioquímica, Faculdade de Farmácia, Universidade do Porto, Porto, Portugal; 2 Departamento de Biologia, Universidade de Aveiro, Aveiro, Portugal; 3 Departamento da Mulher e da Medicina Reprodutiva, Serviço de Obstetrícia, Centro Materno-Infantil do Norte Dr Albino Aroso, Centro Hospitalar Universitário do Porto, Porto, Portugal

## Abstract

Proliferation, differentiation and apoptosis of trophoblast cells are required for normal placental development. Impairment of those processes may lead to pregnancy-related diseases. Disruption of endoplasmic reticulum (ER) homeostasis has been associated with several reproductive pathologies including recurrent pregnancy loss and preeclampsia. In the unfolded protein response (UPR), specific ER-stress signalling pathways are activated to restore ER homeostasis, but if the adaptive response fails, apoptosis is triggered. Protein kinase RNA-like endoplasmic reticulum kinase (PERK), inositol-requiring enzyme 1 (IRE1) and Activating transcription factor 6 (ATF6) are central players in UPR and in ER-stress-induced apoptosis, as well as downstream transcription factors, as C/EBP homologous protein (CHOP). Our previous studies have shown that the endocannabinoid 2-arachidonoylglycerol (2-AG) modulates trophoblast cell turnover. Nevertheless, the role of ER-stress on 2-AG induced apoptosis and cannabinoid signalling in trophoblast has never been addressed. In this work, we used BeWo cells and human primary cytotrophoblasts isolated from term-placenta. The expression of ER-stress markers was analysed by qRT-PCR and Western blotting. ROS generation was assessed by fluorometric methods, while apoptosis was detected by the evaluation of caspase -3/-7 activities and Poly (ADP-ribose) polymerase (PARP) cleavage. Our findings indicate that 2-AG is able to induce ER-stress and apoptosis. Moreover, the eukaryotic initiation factor 2 (eIF2α)/CHOP pathway involved in ER-stress-induced apoptosis is triggered through a mechanism dependent on cannabinoid receptor CB2 activation. The results bring novel insights on the importance of ER-stress and cannabinoid signalling on 2-AG mechanisms of action in placenta.

## Introduction

Placental development comprises tightly controlled processes of proliferation, differentiation and apoptosis of the trophoblast cells by the interplay of hormones, growth factors and other signalling mediators. The endocannabinoids (eCBs) anandamide (AEA) and 2-arachidonoylglycerol (2-AG) may play a crucial role in these events and modulate the complex network of cytokines and hormones in reproductive events such as decidualization, implantation and labour (Brents 2016). The cannabinoid receptor type 1 (CB1), the cannabinoid receptor type 2 (CB2), the main eCBs and their metabolic enzymes, that constitute the endocannabinoid system (ECS), have been reported in first trimester and term placenta and in the trophoblastic type BeWo cell line (Kenney *et al.* 1999, Park *et al.* 2003, Helliwell *et al.* 2004, Habayeb *et al.* 2008, Trabucco *et al.* 2009, Marczylo *et al.* 2010). Besides that, the importance of ECS in placental tissues has been demonstrated in several studies with knockout mouse models. In mice CB1^−/−^, trophoblast cells present reduced proliferation and placenta has a lower weight, when compared with WT mice (Sun *et al.* 2010).

The tight regulation of eCBs levels throughout the menstrual cycle, decidualization and gestation is well known. Alterations in the ECS homeostasis can lead to abnormal modulation of fundamental cellular processes involved in reproductive pathologies, such as preeclampsia, miscarriage and endometriosis. In women with endometriosis, there is a significant increase in plasmatic AEA and 2-AG levels. High AEA levels have also been shown to be related to failure in the *in vitro* fertilization embryo transfer and spontaneous miscarriage, while women with preeclampsia exhibit reduced levels of AEA (Maia *et al.* 2020). Moreover, we previously demonstrated that AEA and 2-AG impair the synthesis of proteins and hormones by the human syncytiotrophoblast (Costa *et al.* 2015*a*, 2016). In addition, AEA and 2-AG are able to modulate trophoblast apoptosis by inducing caspase activation and reactive oxygen species (ROS) generation (Costa *et al.* 2014*a*,*b*, 2015*b*).

Over the last years, placental stress has been linked to the pathophysiology of pregnancy complications such as preeclampsia and intrauterine growth restriction (IUGR), in which oxidative stress and endoplasmic reticulum stress (ER-stress) have gained attention (Burton *et al.* 2017). In fact, it was reported the association between impaired ER homeostasis and reproductive pathologies including endometriosis, recurrent pregnancy loss, preeclampsia and gestational diabetes (Fu *et al.* 2015, Yung *et al.* 2016. Guzel *et al.* 2017). Moreover, ER-stress has been implicated in cyclic endometrial regeneration and remodelling, folliculogenesis, fertilization, pregnancy and parturition (Schoots *et al.* 2018).

The ER contributes to the protein production and folding, storage and regulation of calcium and synthesis and storage of lipids. Therefore, ER is inextricably linked to the maintenance of cellular homeostasis and cell fate decisions (Almanza *et al.* 2019). The ER copes with the burden of unfolded proteins or misfolded proteins in its lumen by activating signalling pathways, collectively known as the unfolded protein response (UPR) (Xu *et al.* 2005, Szegezdi *et al.* 2006). Transmembrane protein sensors located in the luminal face of the ER membrane are activated through dissociation of the ER chaperone glucose-regulated protein 78 (GRP78/BiP). Protein kinase RNA-like endoplasmic reticulum kinase (PERK), inositol-requiring enzyme 1 (IRE1) and Activating transcription factor 6 (ATF6) are ER-stress transducers that sense the protein folding status and transmit the information to the cytosol and nucleus (Xu *et al.* 2005, Szegezdi *et al.* 2006). These proteins enroll UPR-mediated pathway, an adaptive mechanism that includes up-regulation of the ER-protein folding machinery, ER-associated protein degradation and inhibition of protein synthesis. However, above a certain threshold, unresolved ER-stress elicits apoptosis (Zhang *et al.* 2019). The players involved in cell death include PERK/ATF4/Eukaryotic Initiation Factor 2 alpha (eIF2α)-dependent induction of the pro-apoptotic C/EBP homologous protein (CHOP) and IRE1-mediated activation of TRAF2 (TNF receptor associated factor 2), which stimulates the ASK1 (apoptosis signal-regulating kinase 1)/JNK (c-Jun N-terminal kinase) cascade and Bax/Bcl2-regulated Ca^2+^ release from the ER. CHOP has been identified as one of the most important mediators of ER-stress-induced apoptosis (Xu *et al.* 2005) acting through different mechanisms. CHOP induces the down-regulation of the cell survival BCL-2 family members and the up-regulation of pro-apoptotic Bcl-2 homology 3 (BH-3)-only proteins that play a key role in mitochondrial-dependent apoptosis (Rodriguez *et al.* 2011). In addition, CHOP activation results in calcium release from ER to the cytosol and ROS production (Zeeshan *et al.* 2016). Another mechanism of action of CHOP is the modulation of the oxidative state, as overexpression of CHOP leads to an exacerbated increase of ROS at the ER (Malhotra & Kaufman 2007).

The cellular adaptive mechanisms, including the ER-stress-induced coping responses, are physiologically important for a normal placental development, since trophoblast cells undergo complex processes of cellular turnover. Bastida-Ruiz *et al.* (2019) showed that the ER-stress response and UPR play a role in syncytialization both in the human trophoblastic cell line model BeWo and in primary cultures of cytotrophoblasts. ER-stress response inhibition leads to a default in syncytialization, associated with alterations in cell survival. We previously described that 2-AG interferes with cytotrophoblast syncytialization through a CB receptor-dependent mechanism (Costa *et al.* 2015*c*). Moreover, in BeWo cells, we found that 2-AG induces trophoblast apoptosis, through mitochondrial membrane potential loss and increase in ROS generation and caspase -3/-7 and -9 activities, suggesting the activation of the mitochondrial pathway (Costa *et al.* 2014*a*). The endocannabinoid 2-AG is therefore implicated in normal trophoblast turnover. However, it is unknown if the ER-stress response and UPR are involved in trophoblast apoptosis induced by cannabinoids and the role of the cannabinoid signalling. Nevertheless, eCBs are able to induce ER-stress in other cell models (Soliman *et al.* 2016, Almada *et al.* 2017).

Alterations in proliferation and exacerbation of trophoblast apoptosis have been associated with placental-related complications (Huppertz *et al.* 2006, Heazell & Crocker 2008). On the other hand, dysregulation of the endocannabinoid levels might lead to adverse pregnancy outcomes, including impairment of implantation, inhibition of decidualization and compromised placentation (Maia *et al.* 2020).

We have thus hypothesized that the UPR is involved in 2-AG induced apoptosis. Therefore, in this study, we investigated the activation of the ER-stress-induced cell death pathway to address the involvement of ER-stress within the signalling network of 2-AG actions in trophoblast cells.

## Materials and methods

Dulbecco’s Modified Eagle Medium F-12 (DMEM/F12), foetal bovine serum (FBS), antibiotic-antimycotic solution (penicillin G sodium, streptomycin sulphate and amphotericin B) and trypsin were from Gibco/Invitrogen Corporation. The endocannabinoid 2-AG and the cannabinoid receptor antagonists AM281 and AM630 were from Tocris Bioscience (Bristol, UK). Percoll was from GE Healthcare and WesternBright™ ECL HRP substrate was from Advansta (Menlo Park, USA). The selective inhibitor of PERK, GSK 2656157 and the ER stress inducers thapsigargin and tunicamycin were from Santa Cruz Biotechnology. All other chemicals were from Sigma-Aldrich Co.

### BeWo cell culture

BeWo cell line is a human choriocarcinoma cell line obtained from the American Type Culture Collection, being a well-accepted cytotrophoblast cell model. Cells were cultured in DMEM/F12 medium supplemented with 10% (v/v) FBS and an antibiotic-antimycotic solution (AB-AM), incubated at 37°C and 95% air/5% CO_2_ humidified atmosphere. For the experiments, cells were seeded in 96- or 6-well plates at densities 1.5 × 10^4^ and 6 × 10^5^ cells/well, respectively. After adherence (12 h), cells were treated with 2-AG (10 µM), in the presence or absence of AM281 or AM630 (1 µM), CB1 and CB2 antagonists, respectively, or in the presence or absence of GSK 2656157 (1 µM), in cell culture medium with 1% (v/v) FBS for 24 h.

### Isolation and primary cultures of human cytotrophoblasts

Term placentas of normal pregnancies (38–40 weeks of gestation), from Caucasian women living in the Porto region, were immediately collected after spontaneous delivery or elective caesarean section, from Centro Materno-Infantil do Norte – Centro Hospitalar do Porto after written informed consent. All the procedures were conducted after the approval of the Ethical Committee of Centro Hospitalar do Porto, Porto. Cytotrophoblast cells (hCTs) were isolated as described previously (Kliman *et al.* 1986, Keating *et al.* 2007). Briefly, after the removal of decidua, the villous tissue was dissected from at least ten different regions of placenta and the major blood vessels were discarded by fine dissection. Then, the tissue was subjected to a chemical digestion in a solution of trypsin and DNAse I. The resulting cells were separated in a discontinuous Percoll gradient at 1200 ***g*** for 10 min. The cytotrophoblasts were seeded and incubated in DMEM/F12 medium supplemented with 10% (v/v) of FBS and antibiotic-antimycotic solution at 37°C in 95% air/5% CO_2_ humidified atmosphere. For the experiments, cells were seeded in 6-well plates at density of 4.5 × 10^6^ cells/well. After adherence (12 h), cells were treated with 2-AG (10 µM), in the presence or absence of AM281 or AM630 (1 µM), CB1 and CB2 antagonists, respectively, in cell culture medium with 1% (v/v) FBS.

### Assessment of intracellular reactive oxygen and nitrogen species

For the evaluation of intracellular reactive oxygen and nitrogen species (ROS/RNS) generation, BeWo cells were seeded in 96-well black plates, treated with 2-AG (10 μM) for 24 h and incubated with the probe 2′-7′-dichlorodihydrofluorescein diacetate (DCDHF-DA) for 1 h at room temperature in the presence or absence of GSK 2656157, a selective PERK inhibitor. Fluorescence, proportional to the cellular levels of ROS/RNS, was measured using the Biotek Synergy HTX Multi-Mode Microplate Reader (Biotek Instruments). As a positive control, phorbol 12-myristate 13-acetate (PMA) (25 ng/ml) was used. The results are expressed in relative fluorescence units (RFU).

### Western blotting

BeWo cells were seeded in 6-well plates at a cell density of 6 × 10^5^ and primary cytotrophoblast cells at a cell density of 4.5 × 10^6^ cells/well and treated with 2-AG for 24 h. In some wells, cells were also pre-incubated for 30 min with CB1 and CB2 antagonists or with GSK 2656157, a selective PERK inhibitor. The ER-stress inducer thapsigargin was used as a positive control. Cell extracts were prepared in lysis buffer (20 mM Tris pH 7.5, 150 mM NaCl, 1 mM EDTA, 1% Triton X-100) containing a cocktail of protease and phosphatase inhibitors (1:100 v/v). Samples (30 µg of protein) were separated by 10% SDS–PAGE and transferred onto nitrocellulose membranes. Membranes were subsequently incubated with antibodies against rabbit-CHOP (1:100; Santa Cruz Biotechnology), rabbit p-eIF2α and eIF2α (1:200, Cell Signaling Technology) and anti-PARP (1:200; Cell Signaling Technology), at 4°C overnight. Membranes were then washed and incubated with peroxidase-conjugated secondary antibody anti-rabbit (1:2000; Santa Cruz Biotechnology) and proteins were detected by enhanced chemiluminescence. The membranes were then stripped and reincubated with anti-β-actin or anti-β-tubulin (1:500; Santa Cruz Biotechnology) as loading controls.

### qRT-PCR analysis

Cells were collected in TRIzol reagent, and total RNA was extracted according to manufacturer’s instructions and quantified in the NanoDrop ND-1000 spectrophotometer (NanoDrop Technologies, Inc., Wilmington, DE, USA). RNA quality was assessed using a bioanalyzer (Experion RNA, Bio-Rad Laboratories). One microgram of RNA was reverse-transcribed into cDNA by using the GRS cDNA Synthesis Mastermix (GRiSP Research Solutions, PT). For quantitative PCR, cDNA was amplified with KAPA SYBR® FAST qPCR Master Mix 2× Kit (Kapa Biosystems, Woburn, MA, USA), according to kit instructions within a MiniOpticon Real-Time PCR Detection System (Bio-Rad Laboratories). The PCR conditions and primer sequences are described in Table 1. The specificity of the amplified PCR product was evaluated by the melting curve analysis. The fold change in gene expression was calculated using 2^−ΔΔCt^ method (Livak & Schmittgen 2001) with the housekeeping genes, *GAPDH* and *B2M*. As both reference genes revealed stability and similar results, for clarity, we presented data calculated by using GAPDH gene normalized to each control group.

**Table 1:** Primer sequences and qPCR conditions used to assess the gene expression of *ATF4*, *HSPA5* and *DDIT3*, *GAPDH* and *B2M* were used as housekeeping controls.

Gene ID	GenBank	Primer sequence (5′–3′)	Annealing temperature	Amplicon length	Melting temperature
*ATF4*	NM_001675.4	Sense: ATCCTGCTTGCTGTTGTTGG Anti-sense: GTTCTCCAGCGACAAGGCTA	61.1°C	88	83.00°C
*HSPA5*	NM_005347.4	Sense: TTCTGCTGTATCCTCTTCACCAGT Anti-sense: TGTTCAACCAATTATCAGCAAACTC	61.1°C	73	78.50°C
*DDIT3*	NM_001195057.1	Sense: TCTCCTTCATGCGCTGCTTT Anti-sense: AGAACCAGGAAACGGAAACAGA	57.0°C	67	80.50°C
*GAPDH*	NM_001289746.1	Sense: CGGGAAGCTTGTGATCAATGG Anti-sense: GGCAGTGATGGCATGGACTG	55.0°C	358	83.50°C
*B2M*	NM_004048.2	Sense: AGCAGCATCATGGAGGTTTG Anti-Sense: AGCCCTCCTAGAGCTACCTG	59.0°C	229	80.50°C

The specific primer sequences for *ATF4*, *HSPA5* and *DDIT3* were obtained from Oslowski and Urano (2011), whereas *GAPDH* and *B2M* primers were in-house designed using BLAST software found on the NCBI website, with the NCBI reported sequences.

### Statistical analysis

Statistical analysis was carried out by ANOVA, followed by the Tukey post hoc-test to make pairwise comparisons of individual means when significance was indicated (GraphPad PRISM v. 6.0, GraphPad Software, Inc.). The results are the mean of at least three independent experiments carried out in triplicate. Data are expressed as the mean ± s.e.m., and differences were considered to be statistically significant at *P* < 0.05.

## Results

### 2-AG effects in ER-stress markers

We have previously reported that 2-AG induces apoptosis in BeWo cells, by a mechanism involving ROS/RNS generation associated with caspase -3/-7 activation (Costa *et al.* 2014*a*). In this work, we further explored the pathways of 2-AG mediated cell death by evaluating the involvement of ER-stress and the expression of several ER-stress markers associated with the ER-stress-induced apoptosis. The choriocarcinoma-derived BeWo cell line constitutes a widely accepted *in vitro* model representative of cytotrophoblasts. These cells are easy to handle and grow in a relatively short period of time surpassing the low availability of fresh tissue samples (Rothbauer *et al.* 2017). Additionally, they express CB receptors and other members of ECS and respond to cannabinoid stimuli (Habayeb *et al.* 2008).

We first analysed 2-AG actions on the ER resident chaperone GRP78 (BiP). Besides assisting protein folding, BiP regulates ER-stress-signalling pathways leading to UPR survival/apoptosis responses, as it controls the activation of transmembrane ER-stress sensors (IRE1, PERK, and ATF6) through a direct binding-release mechanism.

**Figure 1:**
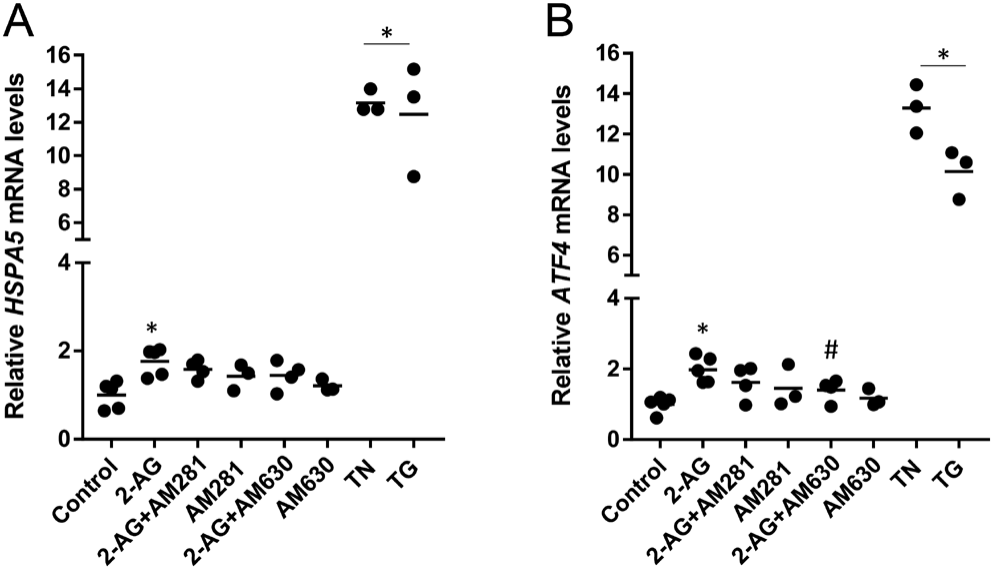
Evaluation of 2-AG effects in ER-stress markers by qRT-PCR analysis. BeWo cells were treated with 2-AG (10 μM) for 24 h. For the assessment of ER-stress dependency on cannabinoid signalling, cells were pre-incubated with the CB1 and CB2 antagonists AM281 and AM630, respectively. (A) 2-AG increased the transcript levels of BiP encoded by the *HSPA5* gene in a CB-independent manner. (B) 2-AG increased the transcript levels of *ATF4* through CB2 activation. This effect was reversed by the incubation with the antagonist AM630, indicating a CB2-dependency. Tunicamycin (TN) and thapsigargin (TG) were used as ER-stress inducers. Results show transcript levels normalised against *GAPDH*. Data are presented as the mean ± s.e.m. (*P < 0.05 vs control; ^#^*P* < 0.05 vs 2-AG).

BeWo cells were treated with 2-AG (10 μM) for 24 h. We observed an increase in the expression of *HSPA5* gene that codifies BiP as a result of the UPR adaptive response. This occurred independently of CB activation (Fig. 1A).

When BIP is released, it induces PERK dimerization and subsequent autophosphorylation. In turn, activated PERK phosphorylates eIF2α leading to global translation arrest, though some transcripts such as ATF4 remain preferably translated. If the stress is persistent, ATF4 can stimulate the transcription of the proapoptotic CHOP to induce cell death. Then, we checked whether long-term activation of UPR by 2-AG may evoke paradoxical response with initiation of apoptotic cell death through the PERK-ATF4-CHOP pathway. In fact, ATF4 was also elevated after 2-AG treatment. This increase was dependent on CB2 signalling engagement (Fig. 1B).

### Cannabinoid signalling modulates 2-AG-induced CHOP expression

**Figure 2:**
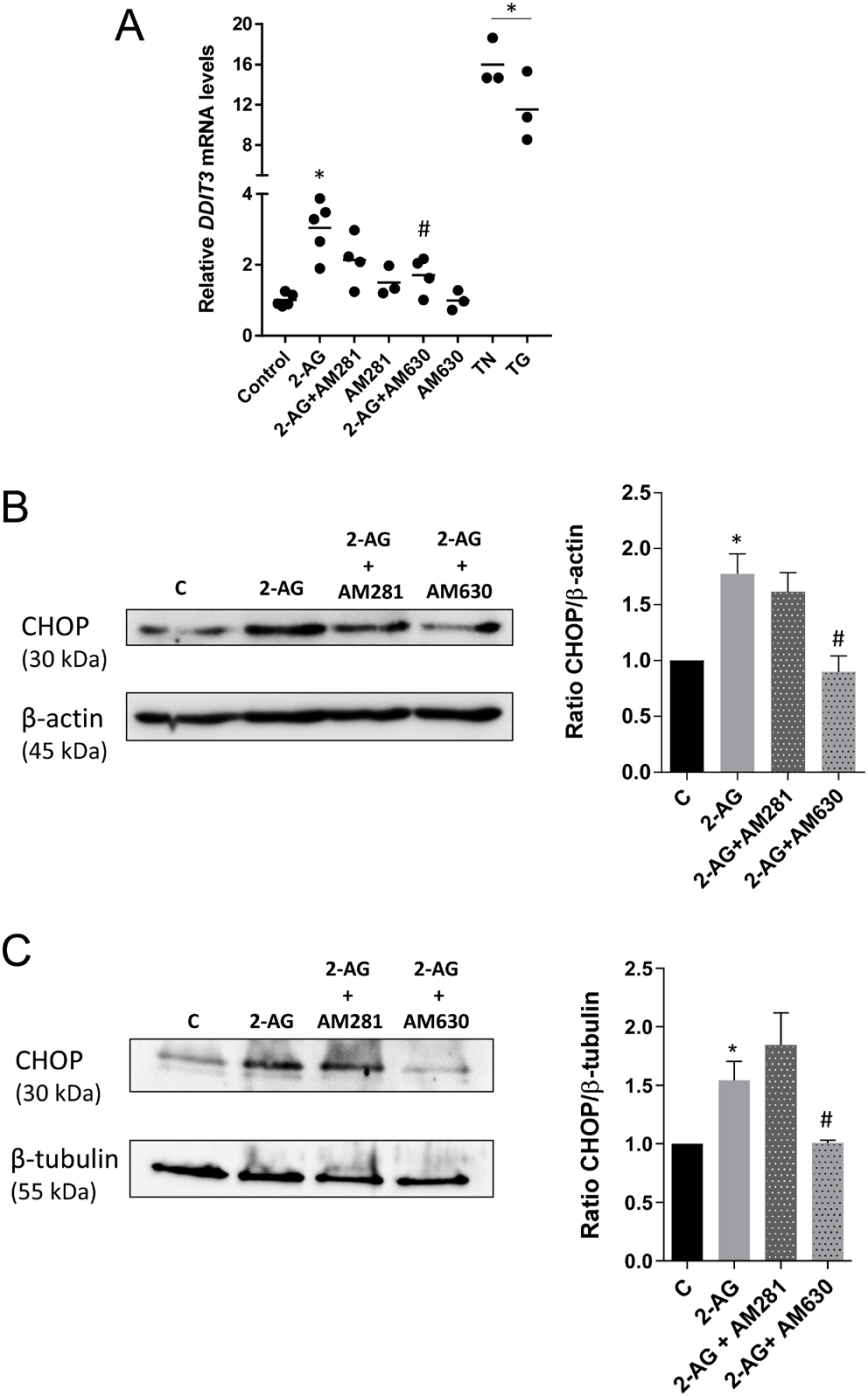
2-AG effects on CHOP expression and modulation by cannabinoid signalling. Cells were treated with 2-AG (10 μM) for 24 h and expression levels of the pro-apoptotic ER-stress factor CHOP were assessed by qRT-PCR and Western blotting. For the assessment of CHOP dependency on cannabinoid signalling, cells were pre-incubated with the CB1 and CB2 antagonists AM281 and AM630, respectively. In BeWo cells, 2-AG treatment increases the transcript levels of CHOP encoded by the *DDIT3* gene (A) and CHOP protein expression (B) through a CB2-dependent manner. Results show transcript levels normalised against *GAPDH*. A representative Western blotting and the densitometry analysis with relative ratios of CHOP/β-actin are shown, as β-actin was used as a loading control. (C) The increase in CHOP protein expression was also observed in cytotrophoblast cells isolated from term placenta and 2-AG effects were also reversed by CB2 receptor blockade with AM631, confirming the involvement of cannabinoid signalling through CB2 activation. A representative Western blotting and the densitometry analysis with relative ratios of CHOP/β-tubulin are shown, as β-tubulin was used as a loading control. Data are presented as the mean ± s.e.m. (*P < 0.05 vs control; ^#^*P* < 0.05 vs 2-AG).

As the prolonged activation of the unfolded protein response may initiate apoptotic cell death via the up-regulation of CHOP, we investigated 2-AG impact on CHOP expression. In BeWo cells, treatment with 2-AG was tested at the mRNA (*DDIT3*) (Fig. 2A) and protein levels (Fig. 2B). After 24 h, we found that expression levels were significantly enhanced with respect to control. The addition of the CB1 antagonist AM281 did not interfere with the expression, while CB2 antagonist AM630 was able to revert 2-AG induced increase.

To further confirm CHOP activation, primary cultures of term cytotrophoblasts were used. Quantification and normalization of Western blotting results (Fig. 2C) showed a similar protein expression profile to the one observed in BeWo cells. Together, these results suggest that the pro-apoptotic UPR response is not only activated by 2-AG treatment but is also dependent on cannabinoid signalling through CB2 activation.

### PERK/eIF2α/ATF4/CHOP pathway involvement in 2-AG-induced apoptosis

**Figure 3:**
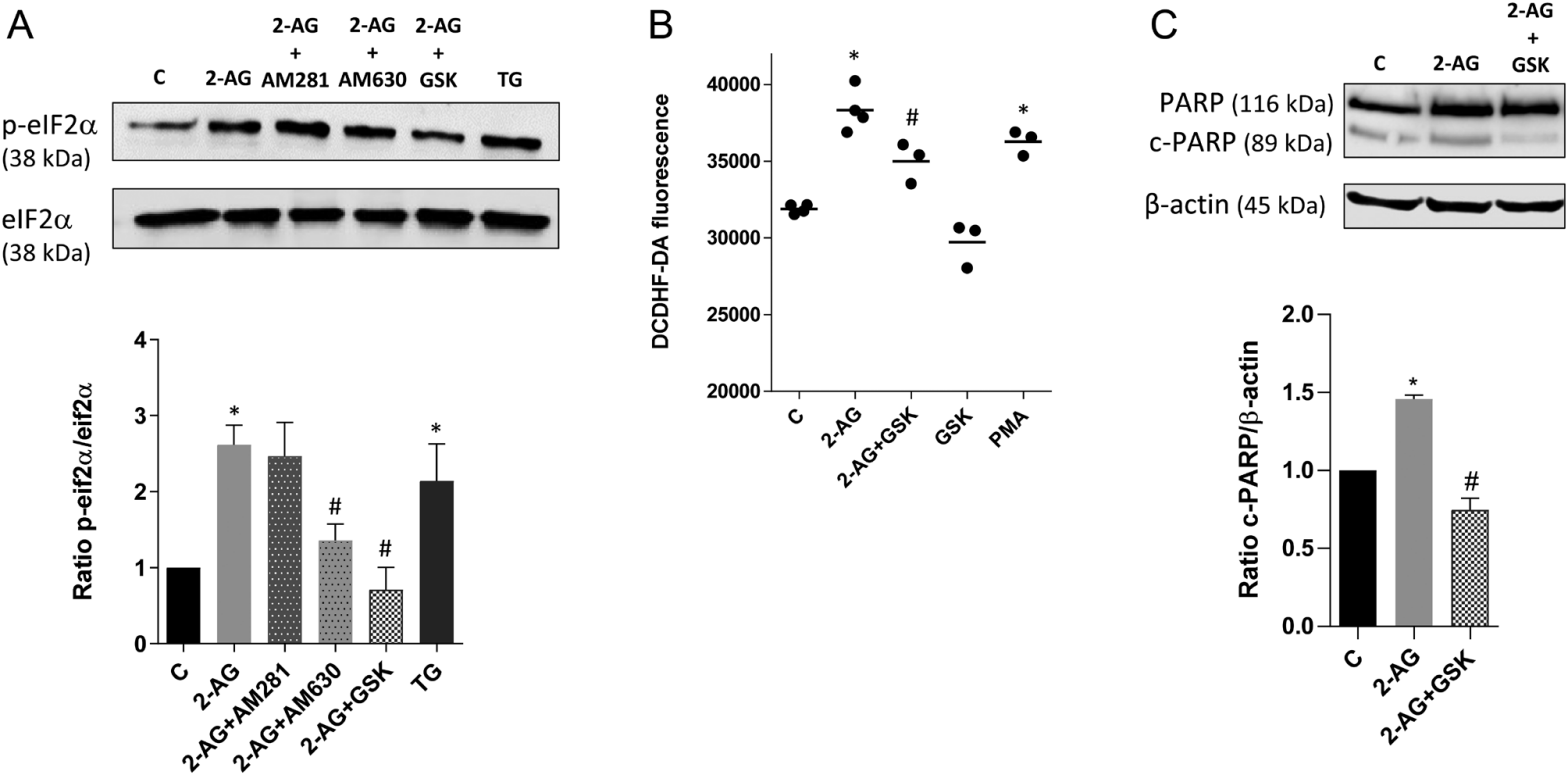
2-AG effects on eIF2α/ATF4/CHOP apoptotic pathway. BeWo cells were treated with 2-AG (10 μM) for 24 h. For the assessment of PERK/eIF2α/ATF4/CHOP apoptotic pathway activation, cells were incubated with the selective PERK inhibitor GSK 2656157 (1 µM). For the evaluation of the dependency on cannabinoid signalling, cells were pre-incubated with the CB1 and CB2 antagonists AM281 and AM630, respectively. (A) Through Western blotting analysis, it was observed an increased phosphorylation of eIF2α, mediated by CB2. The selective PERK inhibitor, GSK 2656157, prevented eIF2α activation. Thapsigargin (TG) was used as a stress inducer. Representative Western blotting, densitometry analysis and relative ratios of phosphorylated eIF2α to total eIF2α are shown. (B) 2-AG induced ER-stress leads to accumulation of ROS. Pretreatment with GSK attenuates ROS generation. Phorbol 12-myristate 13-acetate (PMA) was used as a positive control. (C) Cleaved PARP was determined by Western blotting analysis. Effects of 2-AG treatment were significantly attenuated by pretreatment with GSK. Ratios of cleaved PARP/β-actin are shown. Data are presented as mean ± s.e.m. Significant differences between control and treated cells are denoted by *P < 0.05 vs control and #*P* < 0.05 vs 2-AG.

In response to ER-stress, phosphorylation of the α subunit of eukaryotic initiation factor 2 (eIF2α) reduces general translation initiation, though selectively it enhances the translation of ATF4, involved in the regulation of redox status of cells and apoptosis. Therefore, increased eIF2α phosphorylation induces the ATF4/CHOP pathway. The compound GSK 2656157 selectively inhibits PERK activity in cells by inhibiting stress-induced PERK autophosphorylation, eIF2α substrate phosphorylation, together with corresponding decreases in ATF4 and CHOP. We observed that cells treated with 2-AG present enhanced eIF2α phosphorylation that is reversed by pre-incubation with the selective PERK inhibitor, GSK 2656157 (Fig. 3A). Moreover, GSK alleviates 2-AG induced ROS/RNS generation (Fig. 3B), pointing to the interdependence between oxidative stress and ER-stress. A crosstalk among different cellular components appears essential to recruit pathways leading to cell death. Results demonstrate an increase in poly(ADP-ribose) polymerase (PARP) cleavage, a characteristic of the apoptotic process, that was attenuated by pretreatment with GSK 2656157, indicating the involvement of the PERK arm of the ER-stress in the apoptotic process (Fig. 3C).

## Discussion

Placental development involves well-coordinated processes of trophoblast proliferation, differentiation and apoptosis, in which eCBs play an important role. Moreover, a dysregulation on this balance may be associated with pregnancy-related disorders and infertility (Cuffe *et al.* 2017). High metabolism and oxidative stress are essential to regulate gene transcription associated to trophoblast turnover, invasion and angiogenesis. However, excessive ROS may harm placental development and lead to miscarriage, preeclampsia and intrauterine growth restriction (Schoots *et al.* 2018). ER-stress/UPR signalling-mediated pathways are involved in a broad range of physiologic events including cell differentiation and survival/apoptosis, migration, invasion and angiogenesis (Guzel *et al.* 2017). In a mouse model of constitutive ER-stress, due to a dysfunctional mutation in eIF2α, a premature differentiation of cytotrophoblast cells was observed (Yung *et al.* 2012*b*), whereas genetic knock-out of IRE-1 pathway led to aberrant placental development and decreased trophoblast proliferation (Iwawaki *et al.* 2009). In addition, it has also been suggested that ER-stress is involved in foetal growth restriction, preeclampsia, low birth weight and recurrent pregnancy loss (Yung *et al.* 2008, 2014, Burton *et al.* 2009, Burton & Yung 2011, Kawakami *et al.* 2014, Guzel *et al.* 2017, Lorenzon-Ojea *et al.* 2019). Interestingly, the levels of p-eIF2α, ER chaperone BiP and CHOP proteins were elevated in pregnancies where the growth restriction was complicated by preeclampsia when compared to normal placentas (Yung *et al.* 2008). Moreover, inhibition of eIF2α phosphorylation is associated with reduced cell proliferation and placental villous trees (Yung *et al.* 2012*a*). Increased levels of ER-stress response genes were also observed in preeclamptic and in early pregnancy loss decidualised endometrium (Loset *et al.* 2011, Lian *et al.* 2011).

The role of the eCBs and the cannabinoid signalling in placental ER-stress is totally unknown, though eCBs are involved in ER-stress-induced apoptosis in other cells. In fact, in human pancreatic tumor cells, CB2 dependent accumulation of ceramide upregulates ER-stress related genes (Carracedo *et al.* 2006). In non-melanoma skin cancer, AEA induces ER stress-apoptosis mediated by CHOP expression and oxidative stress (Soliman & Van Dross 2016). Moreover, AEA-oxidative metabolites activate ER-stress apoptosis in tumorigenic keratinocytes through engagement of CHOP and PERK, IRE1 and ATF6 arms of UPR (Soliman *et al.* 2016). Recently, it was reported that the phytocannabinoid Δ^9^-tetrahydrocannabinol, the main psychoactive compound in *Cannabis sativa*, also induces ER-stress in BeWo cells, involving an up-regulation of CHOP levels through the eIF2α/ATF4/CHOP pathway (Lojpur *et al.* 2019).

In this work, we investigated if the endocannabinoid 2-AG was implicated in placental ER-stress and apoptosis, as we have previously reported that, in BeWo cells, 2-AG was able to induce the production of ROS and caspase -3/-7 activation (Costa *et al.* 2014*a*). Here, we demonstrate that 2-AG, via CB2 signalling, leads to the ER-stress apoptotic cell death through the PERK-ATF4-CHOP pathway activation.

We observed that the transcript levels of *HSPA5* gene that codifies BiP were increased in response to 2-AG treatment, independently of CB activation. Nevertheless, the observed alterations in *HSPA5* mRNA expression may also indicate an activation of ATF6 arm of UPR (Mizuuchi *et al.* 2016) besides the PERK-ATF4-CHOP pathway. The release of BiP from stress sensors initiates the transduction of the UPR signals. Although the regulation of ER-stress response at both mRNA and protein level contributes to the overall change in the system (Cheng *et al*. 2016), we decided to study BiP expression only at the mRNA level. In fact, elevated mRNA levels encoding BiP are considered a sensitive and early indicator of ER-stress and have been observed in diseases linked to ER-stress and apoptosis (Ni & Lee 2007, Kroeger *et al.* 2012). In human SH-SY5Y neuroblastoma cells, the ER-stress inducer tunicamycin increased protein and mRNA levels of BiP, as a protective response, while prolonged treatment resulted in apoptotic cell death and up-regulation of CHOP (Reimertz *et al.* 2003). If UPR-induced mechanisms fail to alleviate ER-stress, both the intrinsic and extrinsic pathways for apoptosis can be activated (Sano & Reed 2013).

In addition, we observed that 2-AG increased mRNA levels of *ATF4* and mRNA and protein levels of CHOP involving CB2 activation. PERK phosphorylates eIF2α, which blocks cell proliferation and reduces mRNA translation, but selectively ensues ATF4 translation, that induces CHOP expression (Xu *et al.* 2005). This eIF2α/ATF4/CHOP pathway has been widely explored and plays a crucial role in cell death, particularly in mitochondrial apoptotic pathway (Szegezdi *et al.* 2006). In B-cell chronic lymphocytic leukemia (B-CLL) cells, ER-stress-induced apoptosis is accompanied by increased BiP and CHOP expression (Rosati *et al.* 2010). In endometrial cells and in endometrial cancer cells, eCB-induced apoptosis was also associated to CHOP up-regulation (Almada *et al.* 2017, Fonseca *et al.* 2018). This is also observed for trophoblast cells, as we demonstrate 2-AG sustained increase in the levels of CHOP and apoptosis activation through CB2 receptor both in BeWo cells and term primary cytotrophoblasts. Although placental samples were obtained from a mixture of cesarean and vaginal births, there was no significant variation between controls, though it was previously described that the labour process strongly activates the UPR/ER stress pathways (Cindrova-Davies *et al.* 2007). The PERK pathway is predominant in CHOP activation. However, the observed increase in CHOP expression may result from the activation of different arms of UPR response that we did not explore. For example, IRE1α upon dimerization and autophosphorylation splices XBP1 mRNA and allows translation of an active transcriptional factor XBP1 (Szegezdi *et al.* 2006). IRE1α can also recruit TNF-associated factor-2 (TRAF-2) and apoptosis signal-regulating kinase-1 (ASK1), which causes phosphorylation of p38 MAPK that is associated with CHOP and c-Jun N-terminal kinase 1 (JNK) activation (Sano & Reed 2013, Redza-Dutordoir & Averill-Bates 2016).

Our findings indicate that the PERK/ATF4/CHOP pathway may be associated with 2-AG-induced apoptosis. Interestingly, this UPR arm has also been linked with early-onset of preeclampsia and uterine growth restriction, through the down-regulation of placental growth factor (PlGF), an important regulator of angiogenesis (Mizuuchi *et al.* 2016). In addition, vascular endothelial growth factor A expression, which is necessary for a correct placenta vessel formation is regulated by the UPR-related proteins IRE1α, PERK and ATF6 (Ghosh *et al.* 2010). Importantly, Bastida-Ruiz *et al*. demonstrated that ER-stress and the UPR are involved in trophoblast cell fusion and syncytialization in early-first trimester, late first-trimester and at term (Bastida-Ruiz *et al*. 2019). Moreover, the induction of ER-stress and the UPR processes accompanies the decidualization (Soczewski *et al.* 2020). Therefore, these are all processes that involve the endocannabinoid modulation and the possible crosstalk between eCBs and ER-stress.

The observed attenuation of ROS levels with the pretreatment with the specific inhibitor of the PERK arm of ER-stress, GSK 2656157, suggests the occurrence of an ER-stress-associated ROS production. Many studies have indicated a crosstalk between the generation of ROS and the ER-stress response (Cao & Kaufman 2014). ROS formation in the ER occurs as a regular by-product of disulfide bond formation oxidative protein folding. Protein misfolding may contribute to oxidative stress and ROS generation (Haynes *et al.* 2004, Eletto *et al.* 2014). When ER-stress-associated ROS production is sustained, apoptosis may be triggered (Tabas & Ron 2011). 2-AG induces an increase in ROS, p-eIF2 and CHOP levels that leads to apoptosis as verified by the increase in PARP cleavage, an effect reversed by the addition of GSK. ER-stress and ROS balance are crucial points for placental development, and exacerbation of these processes may lead to pregnancy-related complications.

**Figure 4:**
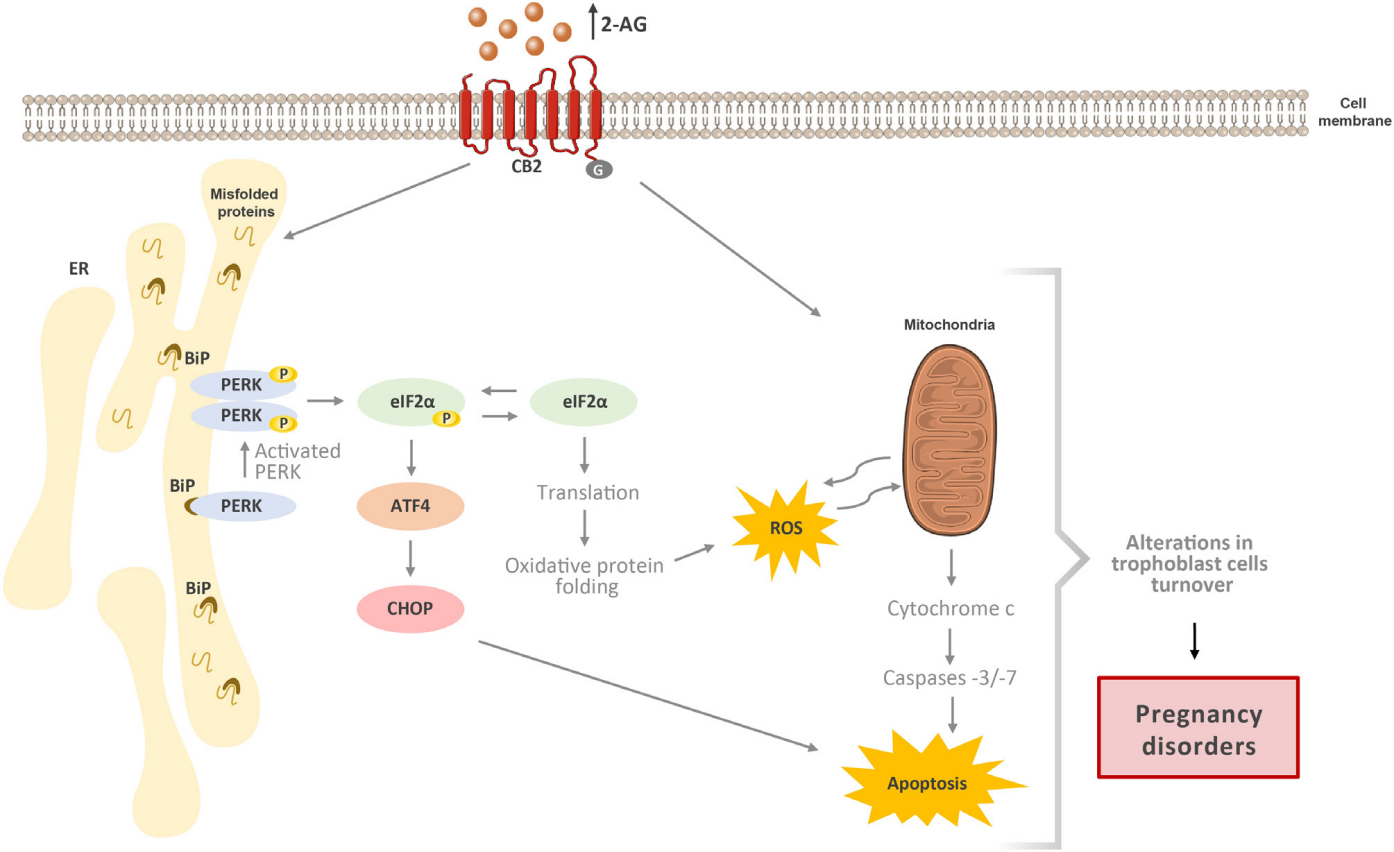
Alterations in 2-AG levels may condition placentation through ER-stress and apoptosis activation: a proposed model. The endocannabinoid 2-AG through cannabinoid receptor 2 (CB2) signalling induces ER-stress and UPR via PERK arm, activating the PERK/eIF2α/ATF4/CHOP pathway leading to ROS generation and apoptosis. Moreover, as we previously reported, 2-AG is also involved in caspase -3/-7 activation and plays a role in trophoblast syncytialization. The signalling pathways involved in ER-stress and UPR play key roles in the normal trophoblast apoptosis and syncytialization. Changes in 2-AG levels/cannabinoid signalling may disturb those processes and therefore the normal trophoblast turnover and promote altered placentation and consequently pregnancy disorders, such as preeclampsia and IUGR.

To the best of our knowledge, this is the first time that it is shown the association of the endocannabinoid 2-AG with ER-stress in placenta. In this work, we propose 2-AG as an ER-stress and apoptotic inducer through the cannabinoid receptor CB2 activation (Fig. 4). These findings along with the antiproliferative effects of 2-AG and AEA on trophoblasts (Costa *et al.* 2014*a*,*b*) further support a crosstalk between cannabinoid signalling, cytotrophoblast turnover and ER-stress that may be implicated in the pathophysiology of some pregnancy complications, such as pregnancy loss, preeclampsia and intrauterine growth restriction.

## Declaration of interest

The authors declare that there is no conflict of interest that could be perceived as prejudicing the impartiality of the research reported.

## Funding

This work was supported by the European Regional Development Fund (ERDF) through the Operational Competitiveness Factors Program – COMPETE and by National Funds through FCT – Foundation for Science and Technology within the scope of the project ‘PTDC/DTP-FTO/5651/2014 – POCI-01-0145-FEDER-016562’. Marta Almada and Bruno Fonseca thank for the grants from national funds and PORTUGAL 2020 Partnership Agreement, HEALTH_RL2_PHD_BIOK_01, NORTE-01-0145-FEDER-000024.

## Author contribution statement

A M, F B M, T N and C G S designed and directed the study. A M, C L and A P performed experiments and analysed data. B J and G D were responsible for sample collection. A M, F B M, T N and C S G wrote and prepared the manuscript. All authors provided critical feedback and helped to shape the manuscript.
